# Gap Junctional Blockade Stochastically Induces Different Species-Specific Head Anatomies in Genetically Wild-Type *Girardia dorotocephala* Flatworms

**DOI:** 10.3390/ijms161126065

**Published:** 2015-11-24

**Authors:** Maya Emmons-Bell, Fallon Durant, Jennifer Hammelman, Nicholas Bessonov, Vitaly Volpert, Junji Morokuma, Kaylinnette Pinet, Dany S. Adams, Alexis Pietak, Daniel Lobo, Michael Levin

**Affiliations:** 1 Center for Regenerative and Developmental Biology and Department of Biology, Tufts University, 200 Boston Avenue, Suite 4600, Medford, MA 02155, USA; Maya.Emmons_Bell@tufts.edu (M.E.-B.); fallon.durant@tufts.edu (F.D.); jennifer.hammelman@tufts.edu (J.H.); junji.morokuma@tufts.edu (J.M.); kaylinnette.pinet@tufts.edu (K.P.); Dany.adams@tufts.edu (D.S.A.); 2 Institute of Problems of Mechanical Engineering, Russian Academy of Sciences, Saint Petersburg 199178, Russia; nickbessonov@yahoo.com; 3 Institut Camille Jordan, UMR 5208 CNRS, University Lyon 1, Villeurbanne 69622, France; volpert@math.univ-lyon1.fr; 4 Octane Biotechnology, Kingston, ON K7K 6Z1, Canada; alexis.pietak@gmail.com; 5 Department of Biological Sciences, University of Maryland Baltimore County, 1000 Hilltop Circle, Baltimore, MD 21250, USA; lobo@umbc.edu

**Keywords:** regeneration, planaria, morphology, head, shape, species

## Abstract

The shape of an animal body plan is constructed from protein components encoded by the genome. However, bioelectric networks composed of many cell types have their own intrinsic dynamics, and can drive distinct morphological outcomes during embryogenesis and regeneration. Planarian flatworms are a popular system for exploring body plan patterning due to their regenerative capacity, but despite considerable molecular information regarding stem cell differentiation and basic axial patterning, very little is known about how distinct head shapes are produced. Here, we show that after decapitation in *G. dorotocephala*, a transient perturbation of physiological connectivity among cells (using the gap junction blocker octanol) can result in regenerated heads with quite different shapes, stochastically matching other known species of planaria (*S. mediterranea*, *D. japonica*, and *P. felina*). We use morphometric analysis to quantify the ability of physiological network perturbations to induce different species-specific head shapes from the same genome. Moreover, we present a computational agent-based model of cell and physical dynamics during regeneration that quantitatively reproduces the observed shape changes. Morphological alterations induced in a genomically wild-type *G. dorotocephala* during regeneration include not only the shape of the head but also the morphology of the brain, the characteristic distribution of adult stem cells (neoblasts), and the bioelectric gradients of resting potential within the anterior tissues. Interestingly, the shape change is not permanent; after regeneration is complete, intact animals remodel back to *G. dorotocephala*-appropriate head shape within several weeks in a secondary phase of remodeling following initial complete regeneration. We present a conceptual model to guide future work to delineate the molecular mechanisms by which bioelectric networks stochastically select among a small set of discrete head morphologies. Taken together, these data and analyses shed light on important physiological modifiers of morphological information in dictating species-specific shape, and reveal them to be a novel instructive input into head patterning in regenerating planaria.

## 1. Introduction

Development, cancer suppression, large-scale remodeling, and regeneration all hinge on an organism’s ability to store and process information about its correct anatomical structure, and correct any deviations from that structure that may occur during injury or other environmental impacts [1,2]. It is commonly assumed that species-specific anatomical shapes are encoded in the genome, although data from environmental epigenetics have long suggested that morphological outcomes are a function of not only inheritance but also of environment and life history inputs [3].

Whether via nucleotide sequences or chromatin modifications, the genome does not specify organismal 3-dimensional shape directly. Instead, large-scale morphology is an emergent feature of the dynamics of complex networks of activity carried out by cells. Thus, pattern formation is the outcome of a rich layer of chemical and physical processes occurring between DNA and anatomy. Indeed, distinct morphologies can arise from a single genotype [4,5]. Understanding how cells communicate and coordinate their functions *in vivo* to reliably form complex body plans and organs is of fundamental importance not only for evolutionary developmental biology, but also for biomedicine [6]. Transformative advances in regenerative medicine and synthetic bioengineering require us to know which inputs can be provided to a cellular system to induce specific morphological outcomes—rational control of growth and form. This is a truly difficult problem because of the complex nonlinearity of biological regulation [7]. Hence, step 1 is uncovering processes that provide instructive control over the determination of large-scale shape.

Most of the field today is focused on gene-regulatory networks [8,9] and physical forces [10,11] in an effort to understand how final patterning outcomes arise and are maintained during development and regeneration. However, another fascinating layer of biological regulation has recently been implicated in the control of morphogenesis: endogenous bioelectrical signaling [12,13,14]. Spatio-temporal gradients of resting potential among all cell types (not just excitable nerve and muscle) can regulate cell proliferation, migration, shape, and apoptosis [15,16]. Even more importantly, they can function as instructive cues for large-scale morphogenesis, regulating positional information, organ identity, size, and axial polarity [1,17,18]. Recent work has implicated these voltage gradients in the regulation of anterior-posterior polarity [19,20], appendage regeneration [21,22,23,24], craniofacial patterning [25], left-right asymmetry [26,27,28,29], eye development [30,31], and brain patterning [32]. Numerous studies have now identified transduction mechanisms linking bioelectric properties with downstream transcriptional and epigenetic targets [13,18,33,34], thus revealing how these physical properties integrate with genetic information during patterning.

Three aspects of bioelectric signaling make them particularly relevant to the origin of large-scale shape and the role of the genome. First, bioelectric patterns specify shape in a distributed (non-local) manner: several studies have shown that the size, shape, and identity of specific structures integrates bioelectrical information from remote regions [32,35,36,37,38,39], making bioelectric signaling an ideal modality for coordinating individual cell behaviors towards a specific anatomical outcome. Second, bioelectric patterns can override default genetic/biochemical information: specific *V*_mem_ changes can prevent mesenchymal stem cell differentiation despite the presence of chemical inducers [40,41], can induce an eye to form in a tissue (e.g., mesoderm or endoderm) that is otherwise not competent to become eye when misexpressing a “master” eye inducer like *Pax6* [31], can induce metastatic melanoma in the absence of genetic damage [36,37], can prevent the formation of tumors in the presence of otherwise-sufficient oncogenes [42,43], and can rescue brain defects caused by mutations in powerful regulators of neurogenesis such as *Notch* [32]. Thus, bioelectric signaling is a good explanatory candidate in instances of epi-genetic influences over pattern formation. Finally, bioelectric properties seem to directly encode outcomes at the level of organs, inducing whole appendages [17,23,24] or complex eyes [31]. This ability to trigger downstream developmental modules, without having to specify individual cell positions (micromanage the process), makes bioelectric signals not only attractive control knobs for biomedical intervention, but also reveals how bioelectric network states can be seen as attractors instructing complex patterning outcomes.

Global bioelectric network dynamics are regulated in part by gap junctions—electrical synapses between cells that facilitate direct ion exchange, thereby allowing cells to compare their *V*_mem_ with those of their neighbors, and establish isopotential cell fields or boundaries between compartments [44,45]. Because gap junctions can be themselves voltage-sensitive [46,47], they in effect can function as transistors—enabling complex information processing and feedback loops. Gap junctions also provide rich opportunities for selective gating of small molecule signals in addition to ion current [48]. Unsurprisingly, because of these features, this versatile signaling element is crucial for development [49,50,51] and also for the plasticity of cognitive memory in the brain [52,53,54]. Thus, we investigated here the role of gap junction-mediated physiological networks in regulating pattern formation.

Planaria are free-living flatworms with impressive regenerative abilities [55,56,57]. After traumatic injury such as amputation, planarians regenerate all missing tissues, reproducing their target morphology perfectly in a relatively short time span of about two weeks. This morphological remodeling is not restricted to trauma; after periods of starvation, planarians shrink themselves allometrically, and grow in the same, scaled manner after food has been reintroduced [58]. This remarkable example of spatial and temporal cellular organization requires the storage and sharing of morphological and physiological information across a diverse and widely dispersed population of cells. Such morphological robustness provides a unique system in which to study complex traits such as the plasticity of morphology. While recent work has made remarkable progress on the molecular details of pathways regulating adult stem cell behavior in planaria [59,60,61], very little information is available on how specific head shape is controlled. The vast majority of the functional literature reports phenotypes of either failure to regenerate or switching of head/tail identity; the field currently has no mechanistic understanding of how a species-specific head shape is established or regulated [62], although voltage-based signaling has been shown to be involved in head shape/size in at least one species [25].

Importantly, different species of planaria have characteristic head shapes that are readily distinguished by anatomical inspection. Thus, we examined the consequences of disrupting the body-wide gap junction communication network for head shape in planaria. Remarkably, we observed a stochastic phenotype in which the regenerating heads of a genomically-normal *G. dorotocephala* flatworm acquired a head morphology appropriate to other extant planarian species. The effect was observed in the head, as well as in the brain morphology. Unlike our recent demonstration of a permanent (stable) change of target morphology in *D. japonica* [20], this effect was temporary, and the worms remodeled back to their native state within 30 days. The ability to stochastically select one of several discrete head shapes appropriate to a different species, simply by altering a physiological network, suggests that quantitative models of bioelectric network modes will be an important part of understanding evolutionary change and the role of genomic *vs.* physiological circuits in establishing anatomical structure.

## 2. Results

### 2.1. Octanol Treatment Induces Changes in Head Shape

**Figure 1:**
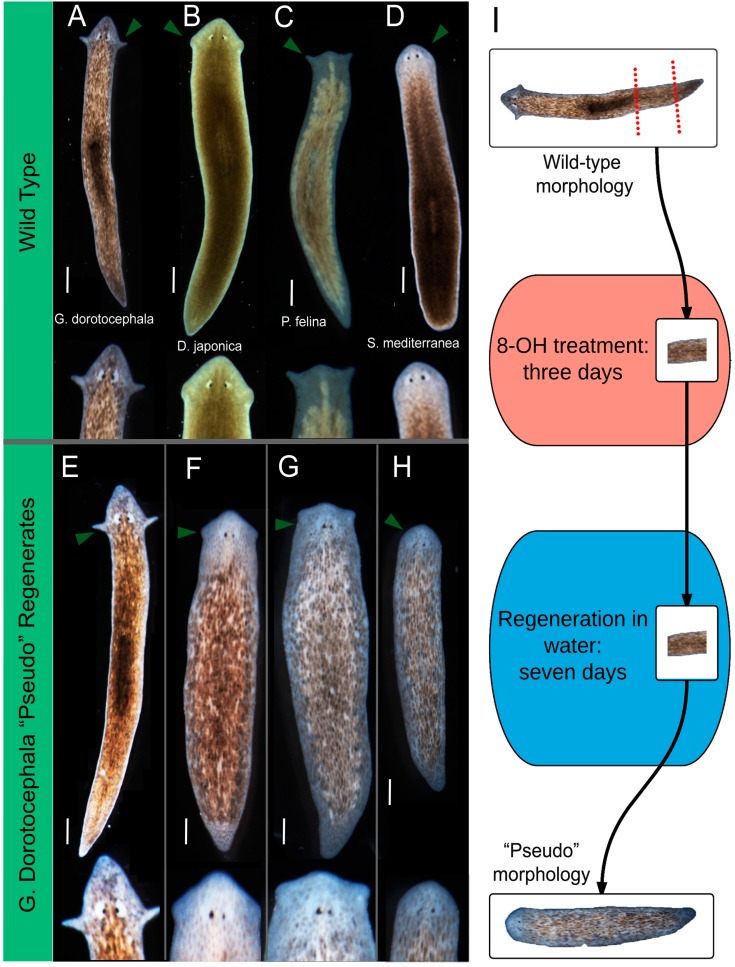
Characterization of varied head morphologies produced by octanol treatment. (**A**–**D**) Wild-type morphologies of four species of planaria flatworm. Arrows indicate auricle placement and general head shape; (**E**–**H**) pre-tail (PT) fragments of *G. dorotocephala*, treated in 8-OH for three days, and then moved into water for the remainder of regeneration (*n* > 243). Arrows indicate auricle placement and general head shape. Scale bar 0.5 mm; (**I**) Experimental scheme of octanol treatment. PT fragments are amputated from *G. dorotocephala* worms. Fragments are treated in octanol (8-OH) for three days, and allowed to regenerate in water for seven days.

Planarian flatworm species display a broad range of head shapes, from the very rounded to the almost triangular, with varied shapes of auricles (Figure 1A–D). To interrogate the mechanisms responsible for regeneration and maintenance of head shape, *G. dorotocephala* planarians were amputated along a plane positioned posterior to the pharynx but anterior to the tail, to produce a pre-tail (PT) fragment. PT fragments were then treated with the gap junction (GJ) communication blocker octanol (8-OH). 8-OH is a commonly-used pan-GJ blocker [20,63,64,65,66,67,68,69,70,71], altering the physiological connectivity between populations of cells, and thereby perturbing the rate and pattern of transmission of bioelectrical and other small molecule signals. Dosage was titrated to a level low enough to enable interference with regenerative signaling without organismic toxicity. 8-OH exposure has been validated to be transient by GC-MS: drug levels are undetectable after a few hours of worm wash-out in water, and octanol does not alter genetic sequences in the worm [20,72].

After 3 days of octanol exposure, worms were allowed to complete regeneration (10 days total). Remarkably, this process resulted in worms that exhibited head shapes highly similar to the heads appropriate to other species of planarians (Figure 1E–H). 8-OH treated fragments regenerated both head and tail correctly, but in many cases, head shape was drastically altered. Wild-type *G. dorotocephala* (GD) possess a very pointed head shape, with two elongated auricles at the plane of the eyes (Figure 1A). Fragments subjected to the same 8-OH treatment scheme regenerated one of: entirely rounded heads like the planarian *S. mediterranea* (SM) (Figure 1H), heads with thick necks and “cat-like” auricles like those of the planarian *P. felina* (PF) (Figure 1G), heads that are triangular like the planarian *D. japonica* (DJ) (Figure 1F), or heads that resemble wild-type *G. dorotocephala* (Figure 1E). We will refer to the head shapes of regenerates as “pseudo” of the morphologically most similar species. Variant, other species-specific head morphologies were never observed after amputation or during regeneration in water (the control condition)—the normal process of head regeneration has 100% fidelity to the species-specific shape (Figure S1A). The same schedule of exposure to hexanol (6-OH), a closely-related compound to octanol which does not effectively block GJs and thus can be used as a control [69], had no effect (Figure S1B). Likewise, intact worms soaked in 8-OH for >3 days did not exhibit any unusual morphological outcomes. Based on these results, we conclude that discrete, species-specific head shapes can be achieved by manipulating the connectivity of physiological networks in the planarian flatworm during head regeneration.

### 2.2. Quantitative Comparison of Induced vs. Genome-Specific Head Shapes

**Figure 2:**
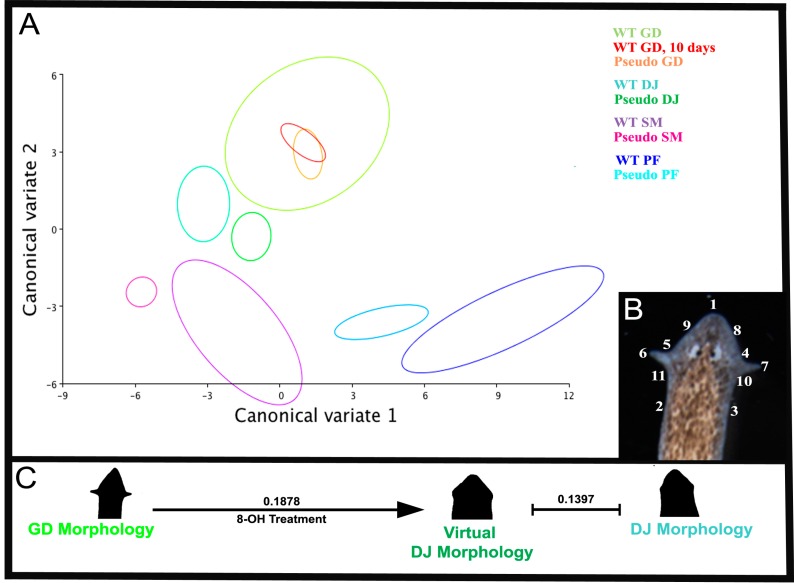
Canonical variate analysis of head shape. (**A**) Graphical output, showing confidence ellipses for means, at a 0.9 probability, of shape data from wild-type and experimentally derived morphologies. Ellipses are colored to correspond with phenotype and treatment. *n* = 10 WT *G. dorotocephala*, *n* = 8 WT *G. dorotocephala* 10 days after amputation and regenerated in water, *n* = 9 WT *D. japonica*, *n* = 6 WT *P. felina*, *n* = 8 WT *S. mediterranea*, *n* = 7 pseudo *G. dorotocephala*, *n* = 13 pseudo *D. japonica*, *n* = 5 pseudo *P. felina*, and *n* = 6 *S. mediterranea* flatworms were measured; (**B**) Legend of landmark placement on a wild-type *G. dorotocephala* head shape (see Materials and Methods); (**C**) Schematic demonstrating alteration of morphology to better resemble another species after 8-OH treatments. Procrustes distances between wild-type *G. dorotocephala*, 8-OH treated *G. dorotocephala* with *D. japonica* head shape, and wild-type *D. japonica* show objective alteration of morphology to be more similar to the non-native species.

Geometric morphometrics [73] was used to quantify similarities and differences between head shapes of true species, as well as the experimentally derived pseudo morphologies. In brief, geometric morphometric analysis involves placement of a series of landmarks, which are both biologically significant and reproducible across all samples, removal of non-shape variation (size, rotation, *etc.*), and performance of a set of statistical analysis [74]. Landmarks were chosen based on the common biological landmarks that existed across samples, and semi-landmarks were placed with prescribed relations to these landmarks (Figure 2B). Landmark data was recorded for *n* > 60 worms, including GD worms whose head shape had been experimentally perturbed by 8-OH, control GD worms who had regenerated in water for 10 days, and adult wild-type worms from each of the three species. Principal components analyses (data not shown) and canonical variate analyses were run on the data set. This enabled visualization of mean shape changes between wild-type species morphologies, and between experimentally derived head shapes. Both analyses resulted in the separation of pseudo morphologies from the wild-type *G. dorotocephala* morphology. Procrustes distances between each of the groups were calculated, in order to produce a quantified metric for comparison of shape differences.

The quantification was used to determine whether the shapes that looked like other species objectively resembled those species, and to suggest a continuous morphospace within which octanol-induced shape change can be visualized along a continuum (from normal to that of a different species). Canonical variate analysis supported the statistical significance of the given pre-defined morphological groupings (in this case, groupings were based on experimental treatment and morphology) (Figure 2A). Comparisons of the Procrustes distances between shape groups (Figure 2A, Table 1) showed that the experimentally derived morphologies were closer in shape to the wild-type morphologies they resembled than the wild-type *Girardia dorotocephala* head morphology. Analysis of variance (ANOVA) of both centroid size and shape between wild-type morphology and pseudo morphology groups also confirmed significant differences between groupings (*F* = 7.94, *p* < 0.0001, and *F* = 7.40, *p* < 0.0001, respectively). We conclude that amputation and treatment in 8-OH can produce regenerated worms whose morphology has changed to become significantly more like that of another species of planarian.

**Table 1:** Quantitative analysis of shape similarities between patterning outcomes and normal head shapes of three species.

Procrustes Distances between Morphologies	WT GD after 10 days in Water	Pseudo GD	WT GD	Pseudo DJ	WT DJ	Pseudo PF	WT PF	Pseudo SM
WT GD	**0.10**	**0.10**						
Pseudo DJ	0.14	0.16	**0.19**					
WT DJ	0.16	0.18	0.15	**0.14**				
Pseudo PF	0.38	0.38	**0.40**	0.27	0.33			
WT PF	0.41	0.39	0.42	0.32	0.37	**0.09**		
Pseudo SM	0.21	0.23	**0.25**	0.09	0.17	0.21	0.26	
WT SM	0.28	0.27	0.24	0.23	0.14	0.31	0.35	**0.22**

Comparison of average Procrustes distances between wild-type, and experimentally-derived altered morphologies. Comparisons of particular interest are shown in boldface. Procrustes distances are larger between wild-type *G. dorotocephala* morphologies and the altered species-specific morphologies produced by 8-OH treatment, than between fragments that regenerated *G. dorotocephala*-resembling heads. In addition, Procrustes distances between 8-OH treated regenerates with altered morphologies, and the species that they resemble, are smaller than distances between regenerates and WT *G. dorotocephala* morphologies. Procrustes ANOVA of centroid size *p* < 0.0001. GD = *G. dorotocephala*; DJ = *D. japonica*; PF = *P. feline*; SM = *S. mediterranea*.

### 2.3. The Head Shape Changes Are Stochastic

**Figure 3:**
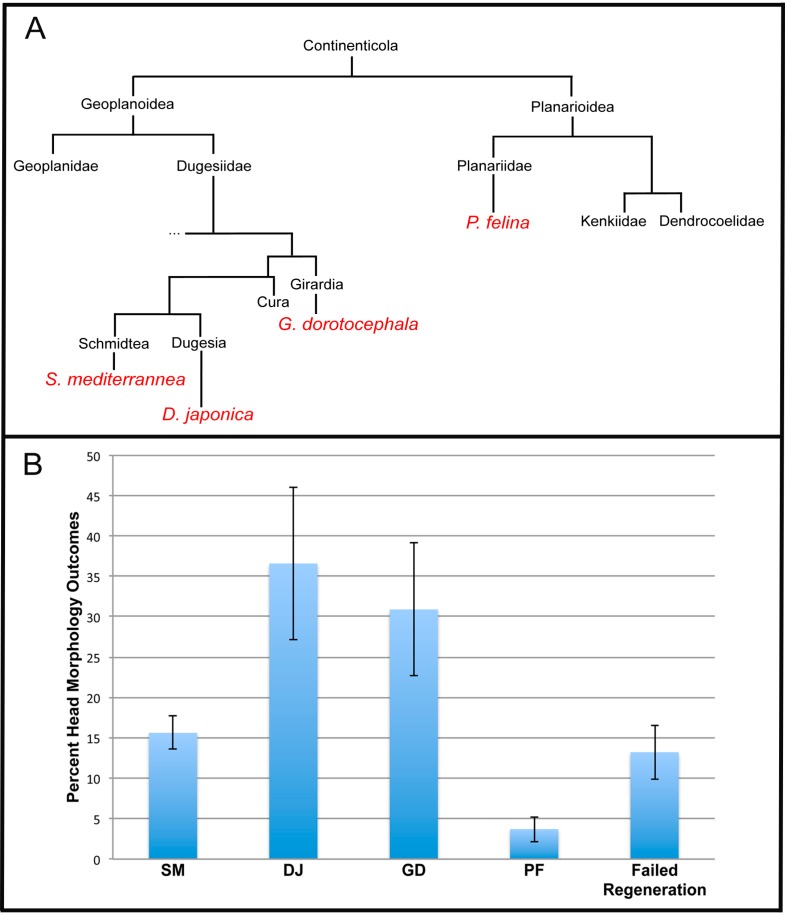
Percentage of head shape outcomes correlates with evolutionary distance. (**A**) Evolutionary tree, constructed from rRNA data, showing relationships between species of interest. Species names in red are those that were analyzed in this work; (**B**) Frequency of head shapes obtained in the octanol exposure experiments (*n* > 243). Failure to regenerate is defined as the loss of anterior-posterior polarity, and the failure to regenerate any head at all after octanol treatment. Error bars are standard deviations.

Obtaining distinct regenerated morphologies, at different frequencies, despite the same treatment conditions, led us to explore the evolutionary relationship between the four species represented. We mapped out an evolutionary tree based on rRNA homology [75,76], and compared this tree (Figure 3A) to frequencies of the different species’ heads arising from 8-OH treatment (Figure 3B). Interestingly, morphologies corresponding to species that are most closely related to *G. dorotocephala* (SM and DJ, which are removed from GDs by at least 100 million years of evolutionary distance) occur with a much higher frequency than morphologies corresponding to less-related species (PF). We conclude that not only are phenotypic outcomes from physiological network perturbation stochastic (as the same treatment leads to one of several discrete shapes among individuals), but the frequencies are not equal and correlate roughly with evolutionary distance between the worm species these heads resemble.

### 2.4. Brain Structure of GJ-Perturbed Worms Is Also Altered to That of Other Species

**Figure 4:**
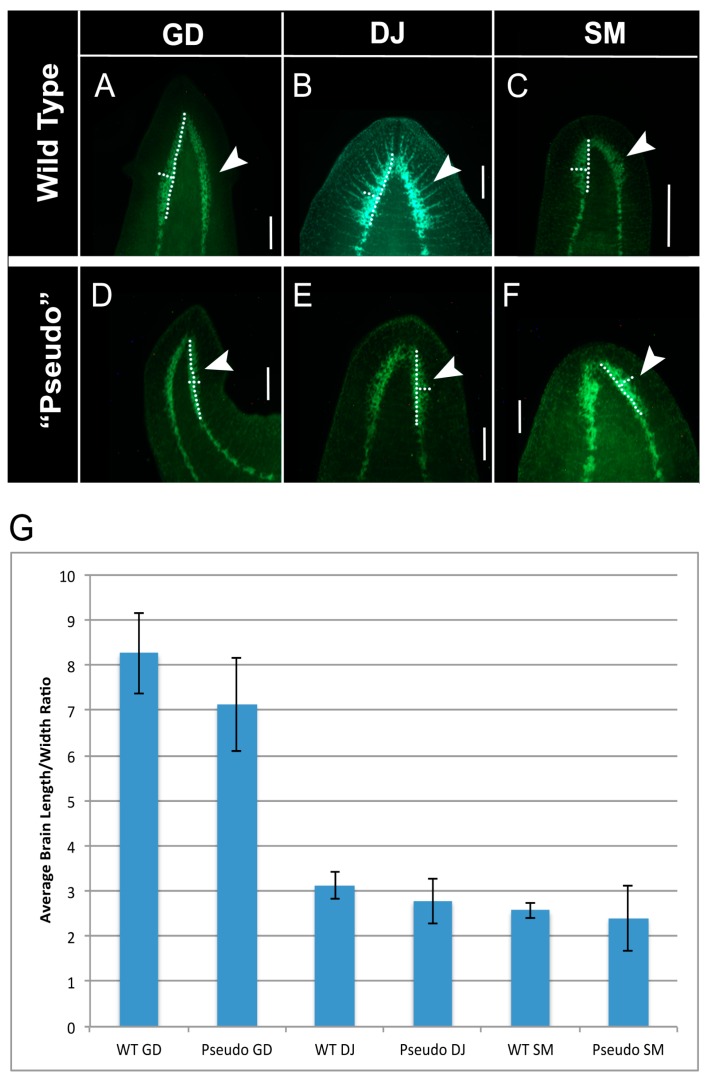
Brain morphology is altered after 8-OH treatment. (**A**–**C**) Brain morphology visualized by anti-synapsin staining of wild-type *G. dorotocephala* (*n* = 10), *D. japonica* (*n* = 15), and *S. mediterranea* (*n* = 6) planarians. Arrows indicate brain morphologies, and dotted lines indicate measurements used for calculation of length/width ratio; (**D**–**F**) Brain morphologies by anti-synapsin staining of *G. dorotocephala* regenerates treated in 8-OH that resembled *G. dorotocephala* heads (*n* = 4), *D. japonica* heads (*n* = 4), and *S. mediterranea* heads (*n* = 6). Arrows indicate brain morphologies, and dotted lines indicated measurements used for calculation of length/width ratio. Scale bar 0.5 mm; (**G**) Average brain length/width ratios of wild-type, and 8-OH treated worms (ANOVA *p* < 4.9 × 10^−14^). Error bars are standard deviations.

We next asked whether internal structures were likewise converted to a different shape, as was external morphology. Few aspects of the planarian internal anatomy differ appreciably between species; however, brain size and shape offer an interesting exception to this rule. The brains of wild-type *G. dorotocephala* are elongated and narrow, while *D. japonica* and *S. mediterranea* have appreciably shorter and wider brain morphologies (Figure 4A–C). No living wild-type *P. felina* could be obtained for this work, and the low frequency of pseudo PF occurrence limited the number available for analysis. Thus, we focused on *G. dorotocephala*, *D. japonica*, and *S. mediterranea*. We performed immunostaining using an anti-synapsin antibody, in order to visualize both the brain, and ventral nerve cords of pseudo and wild-type worms. As recapitulation of wild-type head shape after ten days of regeneration in water had been confirmed by geometric morphometrics, we chose to compare pseudo morphologies to adult worms of other species, in order to minimize confounding data due to variability of regeneration time between species. Overall shape differences were captured by measurement of the brain length/width ratio. These calculations were used to quantify shape differences between species, and to quantify brain remodeling in “pseudo” worms (Figure 4G). Strikingly, we found that pseudo worms possessed brain morphologies that look like the brain morphologies of wild-type worms whose head shapes they resembled (Figure 4D–F) (ANOVA, *p* < 0.001). We conclude that the patterning processes that are disrupted after gap junction communication perturbation are also responsible for producing the morphology of the brain, and that the altered shapes are not limited to the overall head geometry but include the patterning of the central nervous system within.

### 2.5. Distribution of Neoblasts Is Altered to That of Other Species in GJ-Perturbed Worms

Planarians derive much of their remarkable regenerative power from a population of heterogeneous adult stem cells, called neoblasts, which comprise the only mitotically active cell population in the body of the flatworm [56,70,77]. We next investigated whether or not the spatial distribution of neoblasts was appreciably different between species of planarians, and whether GJ-inhibited worms acquired the neoblast distribution characteristic of the species whose morphology they had taken on. As neoblasts are the only mitotically active cells within the planarian body, we performed immunostaining of phosphorylated histone H3, a standard neoblast marker in planaria [78], in order to visualize neoblast populations.

**Figure 5:**
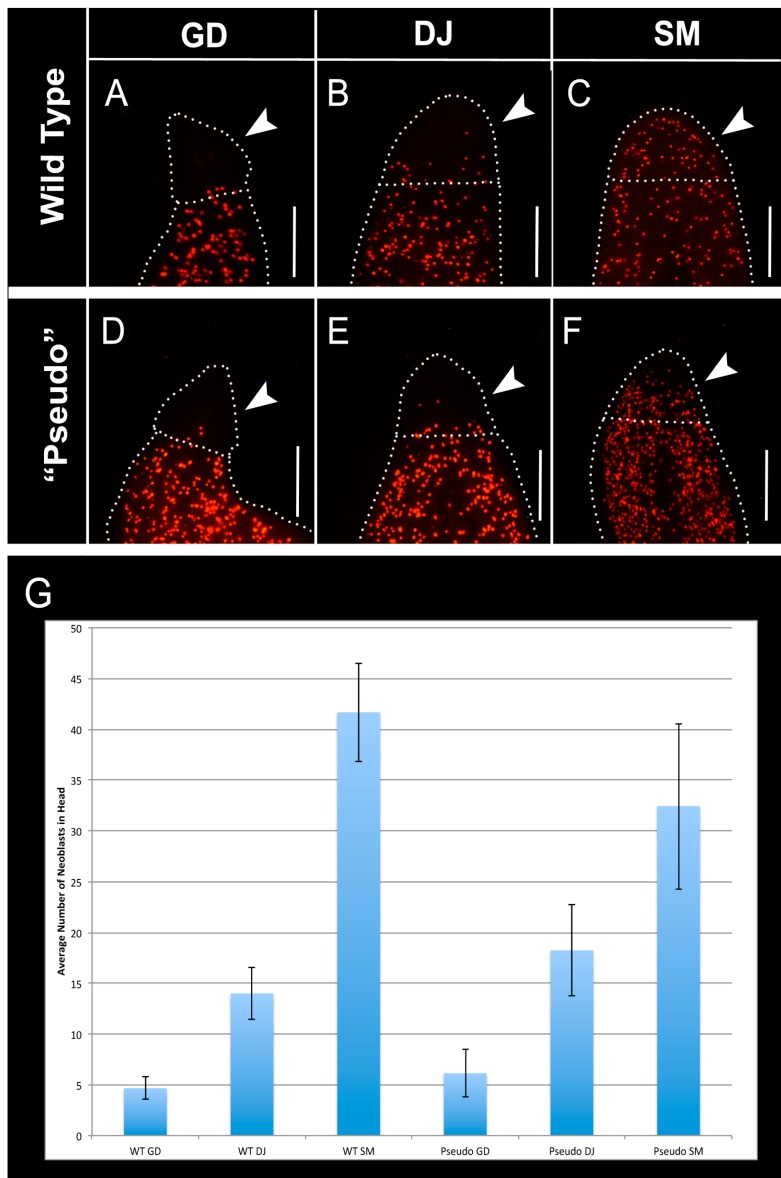
Neoblast (red dots) distribution is altered after octanol treatment. (**A**–**C**) Analysis of neoblast distribution by staining of phosphorylated histone H3 in adult, wild-type *G. dorotocephala* (*n* = 9), *D. japonica* (*n* = 9), and *S. mediterranea* (*n* = 9) planarians. Dotted lines indicate edges of worm anatomy, as well as 1/6th of the length of the worm body from the anterior tip of the worm. This distance was used to define the posterior boundary of the head. Arrows indicate the region in which neoblasts were counted; (**D**–**F**) Analysis of neoblast distribution in *G. dorotocephala* regenerates treated in 8-OH that resembled *G. dorotocephala heads* (*n* = 7), *D. japonica heads* (*n* = 8), and *S. mediterranea* heads (*n* = 7), by anti-phosphorylated histone H3 staining. Arrows indicate region in which neoblasts were counted. Scale bar 0.5 mm; (**G**) Average number of neoblasts in the anterior 1/6th of wild-type, and 8-OH treated worms (ANOVA *p* < 0.05). Error bars are standard deviations.

In wild-type *G. dorotocephala*, very few neoblasts reach into the most anterior 1/6th of the worm (Figure 5A). In wild-type *D. japonica*, the number of neoblasts in the anterior portion of the body is increased in comparison to *G. dorotocephala*, however it is still relatively low (Figure 5B). Wild-type *S. mediterranea* planarians have an abundant neoblast population in the anterior-most region (Figure 5C). All neoblasts in the anterior 1/6th of the worm’s anatomy were counted by hand (Figure 5G). Remarkably, the distribution of neoblasts in pseudo worms mirrored precisely the distribution of neoblasts in the wild-type species that they resembled (Figure 5D‒F) (ANOVA *p* < 0.05). We conclude that the transformation of *G. dorotocephala* worms to resemble other species also extends to the species-specific, characteristic internal distribution of their stem cells, and that the patterning processes that are disrupted after GJC perturbation are also responsible for organizing the distribution of mitotically active cells.

### 2.6. Patterns of Endogenous Relative Membrane Potential Are Altered in GJ-Perturbed Worms

**Figure 6:**
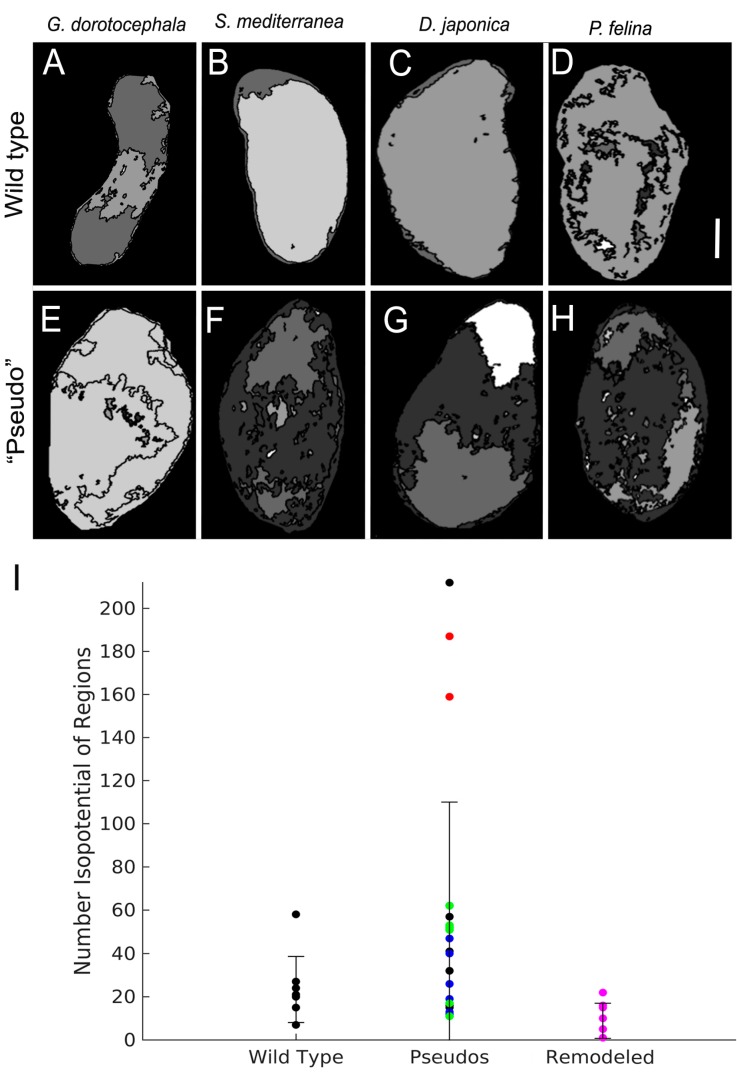
Membrane voltage reporter assay demonstrates long-term change of bioelectrical connectivity in octanol-exposed planaria. (**A**–**D**) Domains of relative membrane potential visualized using DiBAC_4_(3) in wild-type *G. dorotocephala*, wild-type *S. mediterranea*, wild-type *D. japonica*, and wild-type *P. feline*; (**E**–**H**) Domains of relative membrane potential visualized using DiBAC(3) in pseudo *G. dorotocephala* (GDs), pseudo *S. mediterranea* (SMs), pseudo *D. japonica* (DJs), and pseudo *P. felina* (PFs), respectively. Scale bar 0.5 mm; (**I**) Number of isopotential regions in wild-type GD worms (control), all pseudo morphologies, and pseudo morphologies that have remodeled back to WT (wild-type) GD morphology after 30 days. After octanol treatment, the number of isopotential regions in pseudo morphologies is increased, but decreases to WT levels after remodeling. Black dots indicate worms with GD morphologies, blue dots indicate DJ morphologies, red dots indicate PF morphologies, green dots indicate SM morphologies, and pink dots indicate pseudo worms that have remodeled their morphologies to resemble wild-type GDs. *n* = 7 wild-type GD, *n* = 5 pseudo GD, *n* = 5 pseudo SM, *n* = 5 pseudo DJ, *n* = 2 pseudo PF, and *n* = 6 remodeled worms. Error bars are standard deviations. Non-parametric statistical analysis was done using a post-hoc comparison of all groups by Kruskal-Wallis test (*p* = 0.0021), and then between groups using a Dunn’s Multiple Comparison test, which showed differences in voltage domain number between remodeled GDs and pseudo morphologies are statistically significant at *p* < 0.05.

One of the physiological signals propagated within GJ-mediated cell networks is electric current: patterns of GJ-dependent connectivity can determine isopotential cell fields [19,25,79], and we observed that octanol indeed increased the number of regions with distinct *V*_mem_ patterns (Figure 6). Because analytical pipelines to read out encoded pattern states from bioelectrical measurements (as has been done for the human brain [80]) do not yet exist, we sought to begin to establish physiological metrics that could reveal permanent changes of the somatic bioelectric network induced by GJ blockade and could distinguish pseudo worms from those with the original (wild-type) morphology. Thus, we next examined the distribution of isopotential cell groups among the different worm shape outcomes, as such groups are established by the function of gap junctions and are a readout of the topology (connectivity) of developmental bioelectrical networks [20,35,39,51,81,82]. Patterns of endogenous relative membrane depolarization and hyperpolarization were visualized using a DiBAC (*bis*-(1,3-dibarbituric acid)-trimethine oxonol) dye [79,83]. DiBAC is anionic, so dye enters cell membranes based on relative degrees of depolarization [84]. Therefore, increased fluorescence indicates regions of depolarization, and decreased fluorescence indicates relative hyperpolarization. Wild-type worms of the species *G. dorotocephala*, *D. japonica*, *S. mediterranea*, and *P. felina*, and GJ-perturbed pseudo worms were imaged with DiBAC dye in order to assess potential differences in relative membrane potential. Images were analyzed with a custom image analysis program (as described in Methods) to determine the number of distinct isopotential regions present in the entire worm. Analysis of worms 10 days after 8-OH treatment (after regeneration was complete, Figure 6A–H) revealed that transient perturbation of gap junction communication alters body-wide patterns of voltage distribution for many days after the end of 8-OH treatment. We detected an increased number of isopotential regions in the pseudo worms compared to the states of control worms; interestingly, the numbers of isopotential regions return to those of a wild-type state after 30 days of morphological remodeling (Figure 6I). Although we cannot be certain that every single cell had been penetrated by the dye, the pattern of isopotential regions and variability among animals suggests that octanol action is stochastic and not 100% effective, only partially disrupting electrical coupling (revealed as regions that differ in *V*_mem_) throughout animals treated in drug. We conclude that octanol exposure alters the normal pattern of *G. dorotocephala* resting potential toward a pattern that persists even after 8-OH is withdrawn and regenerative repair occurs, producing an increased concentration of isopotential regions throughout the worm.

### 2.7. Induced Shape Changes Are Not Permanent

**Figure 7:**
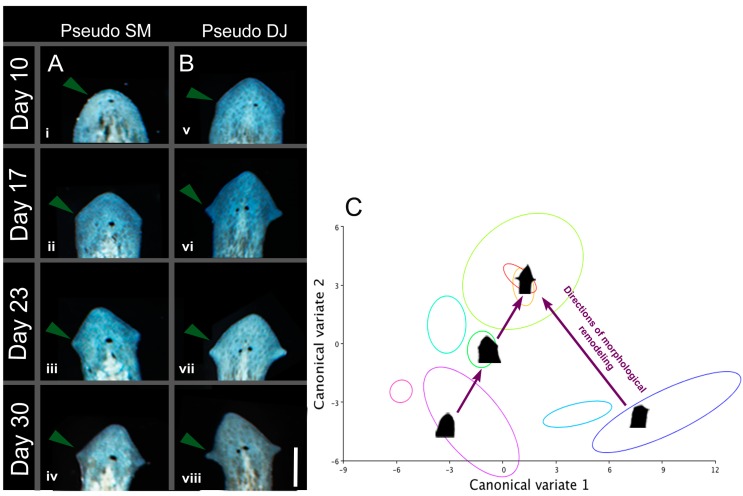
Morphology is remodeled over time to resemble native head shape. (**A i**–**iv**) *G. dorotocephala* treated in 8-OH, which regenerated a pseudo *S. mediterranea* head, tracked over 30 days in water. Arrows indicate auricle elongation; (**B v**–**viii**) *G. dorotocephala* treated in 8-OH, which regenerated a pseudo *D. japonica* head, tracked over 30 days in water. Arrows indicate auricle elongation. Scale bar 0.5 mm; (**C**) Schematic demonstrating “paths” of morphological remodeling through the shape space defined in Figure 2.

In *D. japonica*, physiological perturbations can stably change the basic architecture of the planarian body-plan, producing two-headed worms that continue to regenerate as two-headed in perpetuity across future rounds of regeneration in plain water [20]. Thus, we next asked whether our observed head shape changes in *G. dorotocephala* were permanent. Photographs of worms after treatment with 8-OH were taken daily, from day 10 (when the morphologies of the worms were scored), through day 30 (the time necessary for complete cellular turnover in the planarian flatworm). We found that the induced morphologies were remodeled, long after regenerative processes had ended, to produce morphologies that bore closer resemblance to wild-type *G. dorotocephala*. Interestingly, the time course and result of this non-regenerative remodeling differed depending on the starting head shapes. Regenerates that had rounded heads, which resemble *S. mediterranea*, began to develop a more triangular head shape by day 17, and by day 30, bore more of a resemblance to *D. japonica* than *S. mediterranea*. Over the same time course, regenerates that had triangular heads, resembling *D. japonica*, developed pronounced auricles, and by day 30 were indistinguishable from wild-type *G. dorotocephala* (Figure 7A,B). These findings are fascinating in two respects. First, the change in morphology in absence of a trauma highlights dynamic and robust mechanisms underlying morphological homeostasis—the anatomical state is remodeled over the long-term from an abnormal configuration existing after regenerative repair was complete. Secondly, the remodeling occurs via “paths” through the shape space illustrated by canonical variate analysis (Figure 7C). DJ morphology lies between GD and SM morphologies in this space, and we observe remodeling that moves from SM morphology to DJ morphology to GD morphology. From these data, we conclude that morphology is both plastic and robust, restoration of the “target” morphology can occur without trauma to the organism, and that the CV shape space is informative in illustrating parameters and boundaries to morphological variation.

### 2.8. A Model of Planarian Head Shapes Arising from Cell Interactions

One of the key challenges facing developmental biology and regenerative medicine is linking large-scale patterning outcomes to the individual activity of cells guided by genetic networks and signaling pathways [2,85,86,87,88,89]. Most of the work in the planarian field deals with anterior-posterior fragment polarity [60,62,90] or stem cell differentiation [57,59,91], and does not address the actual morphology of the head or the rest of the body. Recent quantitative, genetically-grounded models of regeneration [92,93,94] likewise use anterior-posterior identity as a binary readout, which does not address or explain changes to the shape of these anatomical regions. Our study of the bioelectrical control of regeneration reported alterations of head remodeling [25], but was limited to scaling and did not model the detailed shape of the head. To begin to mechanistically link individual cell behaviors (such as those regulated by GJ-mediated signals) to large-scale anatomical outcomes, we next constructed an agent-based model of cell signaling and morphogenesis. Our model focused on cell migration and cell-cell signaling, as these are clearly important for implementing different morphogenetic outcomes [95,96,97], and also known to be regulated by GJ connectivity [98] and bioelectric properties of neighboring cells [99].

**Figure 8:**
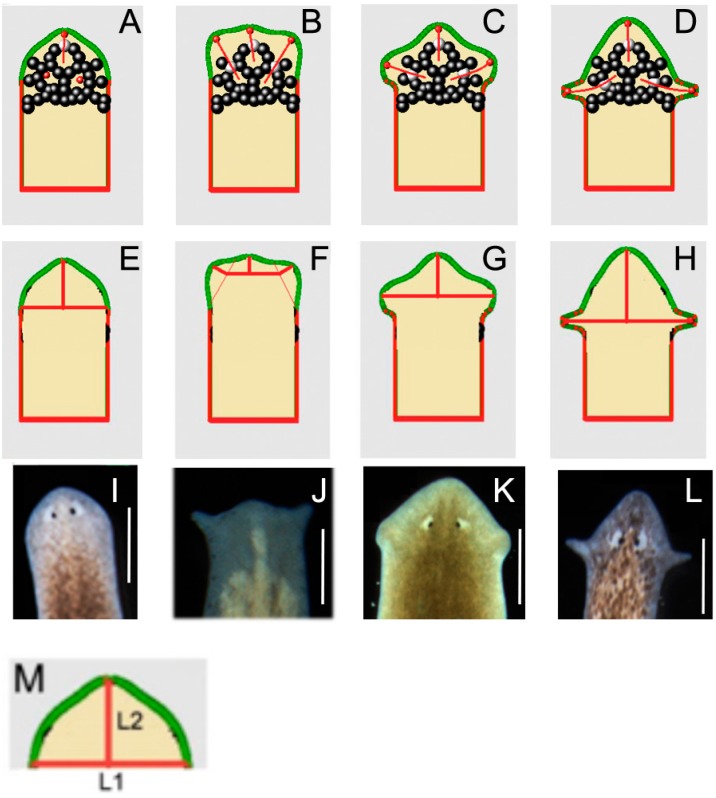
Computational model reproduces the four discrete outcomes observed experimentally. (**A**–**D**) Four types of planarian heads obtained from the computational model. Different shapes result due to deactivation of different cells. Red lines show the trajectories of the red cells; (**E**–**H**) Morphometric measurement of different planarian heads, for comparison with real heads (**I**–**L**) measured (see Table 2). Scale bar 0.5mm. Cells of type A are shown in black, deactivated cells of type A white, cells of type B are red. The outer boundary has two parts differing by its rigidity (soft part of the boundary is shown in red, more rigid part in green). Thus, the red cells represent migrating neoblasts (or their progeny) while the black cells represent somatic cells interconnected by GJs. (**M**) Definitions of lengths of measurements L1 and L2, used in comparison of real worms to those produced by the model in Table 2.

We considered two cell types, A and B. Cells of type A have fixed positions—they cannot move. They produce a substance whose concentration C spreads in space. Its distribution can be described by a reaction-diffusion equation or by some other models. Cells of the type B can move. They do not produce any substance but they receive the substance C produced by cells A and their motion is determined by its concentration distribution. We modeled the planarian head with the following elements: cells of type A, deactivated cells of type A, cells of type B, and a surface boundary. Deactivated A cells are fixed but they do not produce substance C. If there is more than one cell B, then they repulse each other in the same way as they are repulsed by cells A. We choose deactivated cells A symmetrically with respect to the anterior-posterior axis, in order to preserve symmetry of outcome (which implies that B cells move in a similar way on the left and right sides). The outer boundary is composed of points and elastic “springs” connecting them. When a cell B approaches the boundary, a repulsive force acts on it from the boundary. This force is proportional to the distance from the particles and from the intervals of the boundary. Full details of the modeling are given in Figure S2. In this model, we hypothesize that instructive (GJ-dependent) signaling occurs from the somatic tissues to the neoblasts or their progeny, to guide the new tissue generation and shaping during regeneration. Thus, the red cells are migrating neoblasts (or their progeny) while the black cells are somatic cells interconnected by GJs (Figure 8). In the model, octanol disruption of cell:cell communication is thus modeled by the deactivation of signaling from a specific cell type.

When simulated *in silico*, this model produced planarian head shapes observed in the experiments. These different shapes were achieved from the same initial cell configuration but different deactivation pattern of cells A and different elastic properties of the boundary. These deactivation patterns correspond to octanol treatments that reduces cell-cell communication among a subset of cells. We hypothesize that this results in deactivation of some of the cells’ signaling. The initial cells location configuration and its resulting equilibrium configuration are shown in Figure S2H,I. Cells of type B remain surrounded by A cells. This configuration is chosen in such a way that after deactivation of some of cells A, cells B either remain inside or they migrate outside and produce one of the required four configurations (Figure S2A–D). Specific head shape emerges because cells B are pushed away by cells A and by other cells B. They escape through the lower concentration levels of substance C left by deactivated cells A, arriving at the outer membrane and changing its shape.

Note that the shape of the auricles in Figure 8D is more pronounced than in cases Figure 8B,C. This corresponds to the experimentally observed shapes. In modeling, this difference is achieved with different properties of the outer boundary, being softer in the lower part (red in the figure) and resulting in sharper auricles. This is consistent with prior work showing that integument integrity (and thus stiffness) is dependent on the correct expression and function of gap junction channels [100]. Figure 8 shows the final positions of cells B and their trajectories after simulating four different deactivation patterns. In order to compare real worm morphological data to the results of our modeling, we carried out morphometric measurements as shown in Figure 8E–H on the outcomes of our *in silico* simulations. We measured the length of the horizontal interval L1 and of the vertical interval L2 (in the case 1b and 2b these intervals necessarily had to not be straight). The results are seen in Table 2, and provide a good concordance with the observed outcomes in real worms (Figure 1). Let us note that the final morphologies of planarian’s head in computer simulations are determined by the initial cell configuration. They differ by locations of deactivated cells (white cells in Figure 8). If these cells are close, then the final head forms are also close. If we compare DJ (Figure 8C) and GD (Figure 8D), then the initial cell configuration and the final morphologies are more close to each other than these two compared with SM (Figure 8A). Moreover, DJ seems to be in between the SM and GD models. It is interesting to note that this ordering corresponds to their genetic distance (Figure 3). We conclude that a small set of cell-level behaviors, such as migration and cell-cell signaling that are known to be regulated by GJC and thus are perturbed by octanol exposure [35,39,49,98,101,102], can quantitatively explain the morphologies we observed in planaria.

**Table 2:** Morphometric comparison between computational model outcomes and real head shape measurements in 4 species.

*In Silico*		*In Vivo*	
Worm Type	L1/L2 Ratio	Worm Type	L1/L2 Ratio
SM	1.79	SM	1.92
PF	5.0	PF	7.40
DJ	2.46	DJ	2.11
GD	1.85	GD	2.06

These are dimensionless quantities, not dependent on the dimensions of the original data L1 and L2. The latter have arbitrary dimensions (actually measured from the screen).

## 3. Discussion

### 3.1. Perturbation of Bioelectric Networks Produces Inter-Species Morphological Change

Our data show that in the planarian flatworm *G. dorotocephala*, outcomes of morphogenesis can be modified by the perturbation of physiological networks. Brief exposure to a well-characterized disruptor of electrical synapses (gap junction blocker) resulted in regenerated head shapes that quantitatively resembled those of another planarian species. We observed head shapes (and internal structures) resembling *D. japonica*, *S. mediterranea*, and *P. felina.* Note that other species exist with head shapes similar to each (for example, 2 other species are known that exhibit *P.* felina-like flattened heads); because these three types are good representatives of each shape class and are available as living specimens for analysis (unlike other members of the same morphological head types), we refer to our phenotypes as pseudo japonica, pseudo mediterranea, and pseudo felina respectively. Future analysis of other planarian species will reveal if these phenotypes are in fact more closely resembling one of the other members in each shape class.

The head morphology alteration effect was instructive, and not merely one of toxicity or a house-keeping defect, because the outcome was not merely disruption of regenerative ability or a generalized mis-patterning (e.g., tumor or other disorganized growth), but rather resulted in one of several coherent (and ecologically-valid) morphologies. No intermediate morphologies were observed, highlighting the discrete nature of the complex patterning outcomes following GJ blocker exposure. The same 3-day treatment with the closely related alcohol, hexanol (6-OH), which does not efficiently block gap junction channels, and subsequent regeneration in water for 7 days, did not produce altered morphologies, consistent with the importance of the GJ-blocking activity of octanol, and not some other generalized effect of long-chain alcohols.

Dramatically-different morphologies can be derived from the same genome [103,104,105]. Mules and hinnies, which differ only in the sex of the respective donkey and horse parents, are quite morphologically distinct, despite almost identical DNA sequences. Toadflax (*Linaria vulgaris*) has a morphological variant produced solely by variations in DNA methylation patterns, and numerous reptilian species will develop morphologically distinct sexual organs in a temperature-dependent manner [106]. Moreover, recent data revealed how alterations in biochemical signaling can select among different species-specific morphologies [103,107]. Here, we identify a novel physiological input into the process by which distinct morphologies can be derived from the same genome: physiological bioelectric networks. The compatibility of multiple morphological outcomes with a single genomic sequence underlies the importance of learning to understand and control the instructive patterning information that is generated by chemical, physical, and bioelectrical processes beyond hardwired gene-regulatory networks.

### 3.2. Geometric Morphometrics Quantifies Planarian Shape Change

Geometric morphometrics is a technique commonly used to quantify and compare biological shapes. We employed landmark-based analysis, which involves the recording of landmark data, the transformation of resulting shapes to remove scaling and rotational noise, and the performance of statistical analyses. Canonical variate analysis (CVA) was employed to compare wild-type morphologies of four species of worm with the corresponding pseudo morphologies, as well as compare wild-type *G. dorotocephala* morphology to all pseudo morphologies. CVA determines the statistical significance of variation between given groupings of samples [74,108]. In this case the groupings corresponded to observed phenotype and experimental treatment. We chose to employ Procrustes distances to quantify “distance” between shapes, as it is a measure of the square root of the summed squared distances between the corresponding landmarks in two shapes, and is commonly used in morphometric studies comparing differing morphologies. The Procrustes distances between pseudo morphologies and the species that they resembled are smaller than the Procrustes distances between the pseudo morphologies and wild-type *G. dorotocephala.* The observation was supplemented with both Procrustes ANOVA of centroid size and shape (*p* < 0.001, and *p* < 0.001, respectively). This demonstrates a significant alteration of morphology, and quantitatively confirms the apparent similarity between the pseudo phenotypes and their wild-type counterpart species.

Interestingly, the centroids of wild-type *S. mediterranea*, *D. japonica*, and *G. dorotocephala* morphology groups (the three most closely related species) all vary along a linear axis through the CV space. This reveals the morphological consequences of variation along a canonical variate. Moving from negative to positive values of canonical variate one correlates with the elongation of the auricles, if they exist (with SMs being at the negative end of the spectrum, and GDs and PFs being at the positive end). Moving from negative to positive values of canonical variate two correlates with movement of the auricles, if they exist, from a very anterior position, to a more posterior one (with PFs being at the negative end of the spectrum, and GDs being at the negative end).

### 3.3. Morphological Change Is Not Just Skin-Deep

Importantly, the patterning changes induced by electric synapse blockade affect the shape of the brain (Figure 4), not only the overall head shape. Worms with pseudo heads that resemble a given species possess brains that resemble that same species, revealing that the functions of this regulatory pathway extends beyond setting the external outline of the head but instead controls the shape of the brain within. These data also suggest a coupling of brain morphology to head shape. Whether brain morphology drives head shape, or whether both are parallel consequences of the same GJC-dependent upstream control mechanism, this finding demonstrates the depth of re-organization of body plan after perturbation of GJC connectivity. Coupling or involvement of the central nervous system in morphogenesis would not be entirely surprising, given past work demonstrating the necessity of long-range neural inputs for correct polarity patterning during regeneration in planaria [20] and vertebrates [109,110].

In addition, perturbation of gap junction communication reorganizes the distribution of the planarian adult stem cells (neoblasts) to spatial patterns appropriate to other species (Figure 5). Together with the change in brain shape, it is clear that the morphological alteration into pseudo morphologies of other species are thorough, involving not only external head shape but also internal organ scaling and positioning of key cell groups. These data reveal the coupling between proliferative stem cells and large-scale morphology, its variability throughout phylogeny, and its control by GJ-dependent signaling. It is possible that control of neoblast distribution is what drives large-scale morphology. Conversely, their spatial distribution may be one of several downstream consequences of the dynamics of a GJC-mediated cellular network.

### 3.4. Bioelectric State of Tissues and Its Stable Alteration in Species-Specific Shape Change

Our results include the first analysis of the effects of GJ modulation on the long-term bioelectric profile of patterning tissues *in vivo*. While large-scale bioelectric gradients have been shown to functionally instruct pattern formation in a number of models [12], including determination of morphollaxis/scaling in *S. mediterranea* [19] and anterior-posterior polarity in *D. japonica* [19], no studies have yet examined patterns of *V*_mem_ distribution in the context of changes in the electrical connectivity of tissues. We found that brief exposure to a GJ blocker, which is known to be washed out of worm tissues within 24 h [20], results in a greater degree of bioelectrical isolation (ability to maintain numerous smaller regions of isopotential *V*_mem_) as late as 10 days after exposure (Figure 6). Although these long-lasting alterations in bioelectrical patterning are not directly caused by octanol remaining within the body of the worm (since 8-OH is known to depart planarian tissues within 24 h [20]), they are initiated by the octanol-mediated disruption of GJC, which alters bioelectric network topology, and results in persistent perturbation of bioelectric state. In this, the effect resembles the known properties of electrical synapses (GJs) in the nervous system, which help implement a kind of Hebbian plasticity that stably alters ionic conductivity among cells after transient electrical modulation [44,111,112,113,114]. Quantitative physiological models of bioelectrical dynamics will need to be developed in order to understand bioelectric state memory [115] and determine why different types of worms have such distinct profiles of regions of isopotential *V*_mem_.

The variability and overall magnitude of the effect imply that the result of 8-OH exposure is only partially effective in shutting down GJC (affecting some percentage of GJs in any animal); this is consistent with the stochasticity of the morphological phenotype (Figure 3). While 8-OH is a commonly-used method for blocking GJC (and in *D. japonica*, produces the same effect as RNAi targeting innexins genes [20]), we cannot conclusively rule out additional targets besides GJs also being perturbed either directly or secondarily following 8-OH exposure. One difficulty with a molecular (gene-specific) approach to GJ shutdown, is that not only would ~13 innexins need to be cloned out of the GD genome (none have yet been characterized), but their RNAi targeting would have to be tested combinatorially. The space of all possible combinations of different innexin-RNAi’s that would have to be tried simultaneously is enormous, because GJ proteins can often compensate for one another. Nevertheless, our past work tested some such combinations (in *D. japonica* [20]), and showed that a simultaneous knockdown of three Innexins by RNAi reproduces the 8-OH phenotype, strengthening the conclusion that phenotypes are indeed resulting from 8-OH’s effect on gap junctions.

A recent study [20] showing that transient gap junctional perturbation induces permanent changes in planarian target morphology raised the question of whether this pattern memory was established by alterations of GJC that remain long after the blocking reagent leaves the tissues, or by GJC states that alter biochemical or transcriptional cell states which are stable after GJC returns to normal. While we cannot rule out additional epigenetic changes that might occur, our data show that even 10 days later, the effects of brief 8-OH exposure are preserved as decreased electrical connectivity in the somatic tissues. The ability of GJC networks to stably perpetuate induced changes in coupling are consistent with the known roles of GJs in memory and synaptic plasticity [44,52,114,116,117,118].

Our analysis did not reveal a simple correspondence between the specific distribution of voltage gradients and morphological outcomes of pseudo worm regeneration, which could have several explanations. First, it is possible that not voltage but a different molecular signal is what traverses the GJ network to determine planarian head shape. GJ-permeant molecules with signaling roles include serotonin [119,120] and other neurotransmitters [121], histamine [122], calcium [123], and others, which will be tested in future studies, especially as fluorescent probes for some of these physiological signals come on-line [124,125,126]. Another possibility is that each morphological state is achieved by multiple possible distributions of voltage; in this case, the apparent variability would mask our ability to detect a specific mapping from distribution to head pattern outcome. The most likely explanation however is that shape outcomes may not be encoded via steady (single-image) distributions but in time-dependent activity, as occurs during information encoding in the central nervous system. In this case, movies of voltage change across time *in vivo* will have to be acquired and analyzed—a significant challenge in the planaria model system. Our analysis of isopotential regions is only a first attempt to mine the information encoded in bioelectric states. Overall, we believe it is very likely that a much more sophisticated analysis method will have to be developed, perhaps akin to what is being used to infer content of neural networks from brain scans [80,127], and applied to a large sample of worm imaging data, to truly crack the bioelectric code in this system. Functional studies in planaria and Xenopus [19,25,128] have demonstrated the relevance of bioelectrical gradients for anterior patterning in general, but new computational approaches will need to be undertaken to demonstrate what spatio-temporal pattern of bioelectric activity is required for each of the patterning outcomes we observed. Uncovering this mapping will be a focus of our efforts for forthcoming studies.

### 3.5. Morphological Remodeling in 2 Phases: Head Shape Change without Traumatic Injury

The pseudo morphologies we observed are not permanent: they eventually remodel, after the first regeneration process is complete, to once again resemble *G. dorotocephala.* In this, they are distinct from the permanent double-headed phenotype induced in *D. japonica*, which represents a stable alteration of the worms’ target morphology following physiological perturbation [20,129]. Thus, in *G. dorotocephala*, repair occurs in two phases: head regeneration following amputation, which completes in the normal timeframe (<10 days) and results in different species’ shapes, and a subsequent much longer remodeling back to the genome-specific *G. dorotocephala* head shape. This latter process occurs in the absence of injury, within a complete head, over several weeks, illustrating the ability of cellular networks to detect deviations from correct shape, even in the absence of trauma or wounding, and to effect remodeling. Interestingly, the same biphasic process occurs in vertebrates: when a salamander tail blastema is transplanted onto a flank, a tail forms at first, but later remodels into a limb in a much slower process [130,131].

Morphology appears to be a homeostatic parameter, one that is consistently assessed and adjusted in order to maintain some “target” morphology. Morphology must first be decided within days after amputation, as it is possible to observe head shape as early as day six post-amputation. In 8-OH treated worms, this mechanism produces incorrect, pseudo morphologies. However, morphology must then be *reassessed* again after this first decision. When the current morphology does not agree with the target morphology, remodeling is initiated sometime between day 10 and day 17. The activity of these remodeling processes after regeneration has been completed is fascinating, and implies that morphology is consistently reassessed and edited, even after large-scale re-organization events have ended. The nature of the processes that drive cell behavior towards a specific, stable end-state (when remodeling ceases) is almost entirely obscure. At this time, it is not possible in any model system to derive specific shape information (to know precisely which anatomical pattern will be a sufficient end-goal state to cause remodeling to stop) from genomic data.

### 3.6. Computational Modeling of Head Shape Formation

The different head morphologies we observed, which are distinct in outward anatomy, distribution of the stem cells, and shape of the brain, must result from differential cell migration and cell-cell communication. To link cell-level behaviors with the discrete large-scale morphologies and explore the consequences of reduced GJ communication (induced by octanol exposure) on emergent cells signaling and morphogenesis, we analyzed an agent-based model. In this model (Figure S2), some cells actively migrate, and alter the shape of the resulting tissues. Their movements are guided by a chemical gradient produced by other static cells in the head, which pattern of activation is established through GJC, as is known to occur in the worm [20,70] and in many other organisms [51,132,133]. Our model shows the morphogenetic consequences of disruption of this communication, which parallels the interplay between signaling of planarian neoblasts (stem cells) and surrounding somatic tissues. In this model, we propose that significant instructive (GJ-dependent) signaling occurs from the somatic tissues to the neoblasts or their progeny, to guide the new tissue generation and shaping during regeneration.

**Figure 9:**
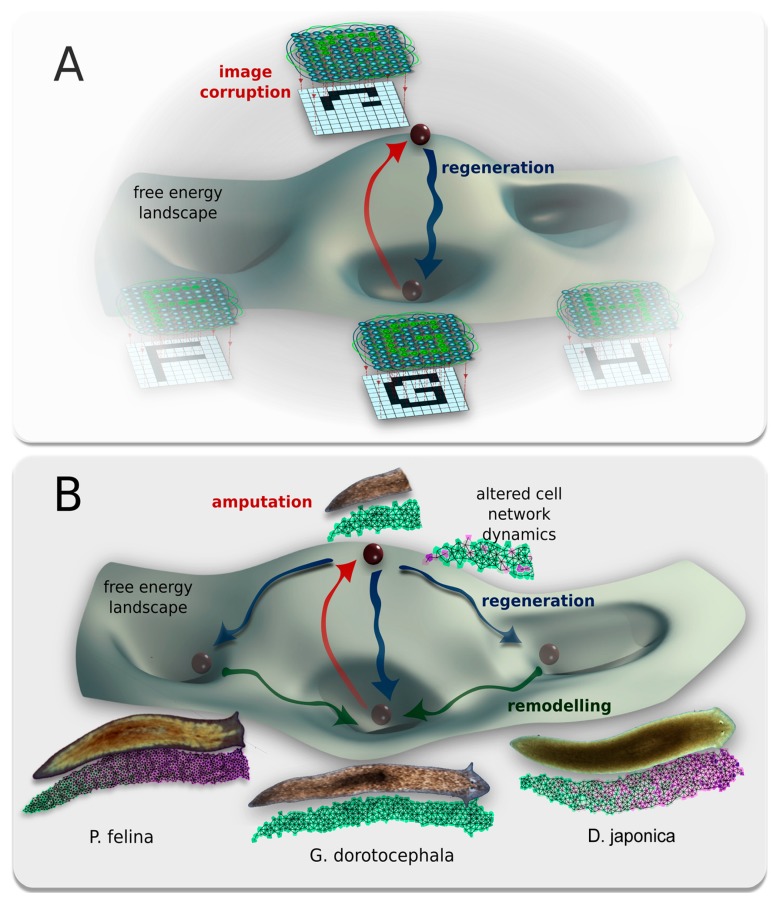
A conceptual model of shape change driven by physiological network dynamics. Planaria regeneration parallels classical neural network behavior; both can be described in terms of free energy landscapes with multiple attractor states. (**A**) Behavior of a classical Hopfield neural network trained to reproduce three types of patterns, in this case shapes of the letters “F”, “H”, or “G”, which are the three stable states of the network’s free energy landscape. The state of the Hopfield network’s nodes directly relate to the brightness of pixels on a display, generating output. Perturbation of the network from a stable state (red arrow) by deleting (damaging) part of the pattern is akin to moving a ball to an unstable point on the free energy landscape, and leads to regeneration of the closest learned attractor state (blue arrow). In this, such networks’ well-known ability to implement memory is analogous to regenerating organisms restoring a specific target morphology upon damage; (**B**) The parallel concept of planaria regeneration into head shapes of one of three different species, which are attractor states of the free energy landscape. Outcome morphology is driven by the dynamic outputs of physiological cellular network. Amputation (red arrow) is akin to moving the system to an unstable point on the free energy landscape. Normal regeneration would return the system to its main attractor, but altering cell network dynamics via gap junction blockade allows for regenerative transitions (blue arrow) to alternative local minima, corresponding to morphospace regions normally occupied by *P. felina* and *S. mediterranea* worms. In time, remodeling (green arrow) transfers these morphologies to the global minimum of the wild-type state (*G. dorotocephala*).

The computational simulations revealed how a small number of cell configurations could result in head shapes quite closely corresponding to the observed morphologies (Figure 9, and Table 2). Interestingly, when specific static cells (black) lose the ability to send their signal due to GJC blockage by octanol, the migrating cells’ movements (red) are altered, giving rise to one of the 4 outcomes. Thus, the model exhibits the two main properties of the experimental dataset: multiple possible outcomes (due to different patterns of GJC-blocked static cells, possibly from physiological noise in pharmacological drug-target interaction), and discrete specific outcomes (resulting from the deterministic signaling of static “black” cells and consequent behavior of migratory “red” cells). It should be noted that our model is also readily applied to an organ that grows from an initial cell position to its final position: if we allow division of cells B, cell migration proceeds in the same way as shown: they will leave behind them deactivated (differentiated) cells B that do not move and do not divide. Future work will test the applicability of this class of models to other regenerating systems.

Our agent-based model includes physical parameters of cells [95,134], as well as traditional chemical gradient diffusion [135,136]. In the model, octanol disruption of cell:cell communication is modeled by the deactivation of signaling from a specific cell type; one candidate for this unique cell population are the rare but widely distributed cells that express the gap junction protein Innexin 5 [71]. Future experiments will test the specific predictions of this model and assign molecular-genetic identities by the several different cell types postulated in the model. An additional important line of investigation is to mechanistically link large-scale dynamics of physiological signaling to the specific behavior of cells modeled in our simulations.

### 3.7. A hypothesis: Physiological Network Attractors Underlie Regions of Anatomical Morphospace

The outcome of regeneration in this model is a set of coherent, discrete head shapes—not a confusing amalgam of different patterns that would be expected if cells made purely local decisions and were merely dis-coordinated by octanol treatment. Currently, the field largely lacks conceptual models for thinking about these kinds of inputs to development and regeneration; it is not clear how best to represent or model discrete, yet stochastically-chosen, emergent large-scale patterns. Yet, this is an increasingly important goal as advances in regenerative medicine and synthetic bioengineering require the ability to predict and control global outcomes by tweaking cell-level parameters. The field of developmental bioelectricity is still searching for an appropriate formalism, but one obvious candidate is that of the neural network paradigm used by computational neuroscientists to understand emergent dynamics of decision-making in brains composed of electrically-coupled cells. We have previously argued that because somatic cells have many of the same components as nerves (ion channels, neurotransmitters, GJs functioning as electrical synapses, gene expression regulated by electrical activity), we may profitably use some of the concepts from neuroscience to understand similar phenomena in slower developmental networks, where output is not muscle-driven behavior but pattern formation [1,13,137].

Scientists in computational neuroscience have modeled the states of electrically-coupled networks in an energy space that reveals the landscape of trajectories that the networks undertake [138,139,140,141,142,143]. Such (neural) networks can stabilize in attractors in this virtual space, and their activity can be parsimoniously explained by networks’ efforts to minimize a global “energy” function [144,145,146,147,148]. Thus, we propose that one way to conceptualize the linkage between bioelectrical connectivity among worm cells (GJC) and discrete global behavioral outcomes achieved by regeneration is to view the worms’ cells as a neural-like network, processing developmental information more slowly but obeying the same statistical dynamics rules as are known to occur in the brain (Figure 9A). It is likely no accident that GJs are well known to be important for information processing in the brain [54,149,150].

We hypothesize that distinct shape outcomes may be attractors in a state space describing distinct modes of the bioelectric network of planarian body cells connected by gap junctions (electrical synapses); in essence, these attractors correspond to regions of a morphospace as classically understood by evolutionary theory [151]. In the experiments, octanol disrupts the normal dynamics of this network, destabilizing and knocking its stochastic dynamics into one of several nearby attractors, normally belonging to different species of flatworm (Figure 9B). Developing a quantitative, computational model of cell networks that have such modes (with electrical states impinging on cell proliferation, apoptosis, and migration behaviors that result in different head shapes) forms the next deep challenge in this area.

Such dynamic networks underlie virtually every complex process known in the biological world, from metabolism to group behavior [152,153,154]. These richly interconnected nonlinear networks, made up of many simple units, have the ability to produce complex pattern in open and thermodynamically unstable systems, without any single governing body [155,156]. Within a given system, these networks converge onto one or more discrete, stable states [157]. Neural networks are just one example of complex dynamic networks; they are implemented via electrical signaling and gap junctions that demonstrate discrete attractors corresponding to global states [141,158], and stable re-occupation of large-scale states (memory) after perturbation [54,147]. Given that neural signaling evolved by specializing much more ancient somatic physiological communication mechanisms, it is reasonable to postulate fundamentally similar network dynamics for physiological networks during pattern formation [137,159]. On this view (Figure 9), the discrete head patterns are outcomes of bioelectric network dynamics with the lowest free-energy; cutting the network raises the free energy by deviating the system from its optimal morphology (damage), and initiates the regenerative process—a journey back down to a low-energy attractor. Perturbing the physiological bioelectric network with 8-OH results in shifting the system dynamics from this attractor to another attractor, and subsequent regeneration of an altered morphology. Apparently, these pseudo shapes are only local minima, since after about 3–4 weeks the pseudo worms return to the global minimum of the standard *G. dorotocephala* morphology.

In this class of models, the output of the physiological network consists of signals directing cells to proliferate, apoptose, or migrate, so that head morphogenesis is achieved. The details of this process at the cellular level is beginning to be understood, both in the CNS (control of gene expression by brain activity [160]) and developmental bioelectricity [12,23,32]. Variations in membrane potential between cell groups may act as electrophoresing forces, pulling small signaling molecules, small RNAs, or charged transcription factors to specific regions of the planarian body, producing numerous possible long-term effects [47,161,162,163,164]. Additional mechanisms include voltage-sensitive phosphatases [165], and voltage-regulated action of chromatin modifying enzymes [34,43,166]. Clearly, this is just one way to think about these data, and others are possible. The advantage of this paradigm is the rich set of mathematical results available from studies of computational neuroscience that can constrain future model-building and help make testable predictions.

### 3.8. Stochastic Phenotypes and Evolutionary Implications

One of the key aspects of these phenotypes is their stochastic nature: the same treatment performed on a cohort of clonal (isogenic) animals results in one of several patterns within any exposed population. Strikingly, the frequencies with which species-specific patterns are observed match the evolutionary distances estimated for these species of planaria (Figure 5). There are significantly more pseudo *G. dorotocephala*, *D. japonica*, and *S. mediterranea* morphologies, which correspond to a smaller evolutionary distance from *G. dorotocephala*, while *P. felina* pseudo morphologies remain relatively infrequent. One possibility is that a core network (composed of gene regulatory elements and physiological gap junction communication (GJC)—mediated signals) is at work among many planarians, with species-specific differences tweaking its key dynamics. The evolutionary distance among the planarian types may thus be expected to reflect the ease of shifting this network into its different potential outcome states (as occurs during GJC inhibition). It should be noted that wild-type *P. felina* have multiple eyes along the doso-ventral body boundary, a trait that our pseudo PFs do not display. Incomplete mimicry of the most distant species, *P. felina*, is consistent with the association between evolutionary distance and ease with which species-specific morphology can be fully recapitulated during perturbed regeneration. It should be noted that *Phagocata gracilis* and *Phagocata velata* planarians also have head morphologies quite similar to *P. felina*, and therefore also to pseudo PFs. These two species possess only two eyes, and both species are similarly evolutionarily removed from *G. dorotocephala*. However, no living wild-type worms of these two species could be procured for this work, and therefore our analysis focused on *P. felina*. Overall, the ability to generate distinct morphologies from the same genome likely represents an extremely flexible mechanism that could be exploited by evolution via many known environmental and genetically-encoded modulators of gap junctional state.

An obvious absence of “intermediate” morphologies, or head shapes that resemble a blending of two or more species of planarian, could be the result of constraints that limit the morphological space available to the regenerating planarian. Within this framework, the constraints on phenotypic space become driving factors in morphogenesis. These constraints are likely an amalgam of developmental, environmental, and evolutionary factors, which serve to limit the stable modes of the patterning network, and thus the morphogenetic possibilities available to a regenerating flatworm. Efforts currently on-going in our lab are aimed at producing mechanistic bioelectrical models of single cells and cell networks to quantitatively explain the discrete stable modes of such networks, the stochastic nature of each “run” of such a network following amputation, and the molecular links to downstream cell behaviors [94].

### 3.9. Limitations and Future Approaches

Our study faced several limitations. First, we have not yet produced cell-resolution spatio-temporal datasets on voltage and GJ connectivity during remodeling, of the kind used to extract semantics of bioelectrical states in brain activity [80,127]. Second, while electric current is a very likely messenger transferred through the gap junctional paths [19,167], involvement of other small molecules such as neurotransmitters, IP_3_, and calcium are also possible mediators of the signaling that regulates cell behavior during head morphogenesis [35,119,120,122,168,169]. All of these questions can be investigated once again-of-function technology (misexpression) becomes available in the planarian model system. In addition, future experiments that could reveal differences among pseudo morphologies would target non-coding small RNAs and chromatin modifications.

It should be noted that any model seeking to explain these data would have to include not only a list of molecular components required for the process, but a constructive scheme exhibiting discrete, stochastic, large-scale morphogenetic outcomes. Thus, the biggest area for future development, in addition to molecular details, is computational modeling of a gap junction-based network with sufficient quantitative detail to reproduce the observed stochastic, discrete attractor modes corresponding to specific morphologies. This effort is currently on-going in our lab [137,170,171]. A truly successful model should explain why these particular shapes are produced, and allow the experimenter to control the patterning outcome at will by appropriate manipulation of physiological state and connectivity—a kind of guided self-assembly, as is becoming possible in the frog model system [1,13,17,33].

## 4. Materials and Methods

### 4.1. Husbandry and Pharmaceutical Treatment

*G. dorotocephala* planaria were obtained from Carolina Biological Supply Company. Worms were kept in commercial natural spring water (Poland Springs water, Framingham, PA, USA) at 20 °C, and starved for >7 days before amputation. Pre-tail (PT) fragments were obtained by making transverse cuts posterior to the pharynx and anterior to the tail, using a sharp scalpel. Worms were immobilized by placement on a moistened and cooled Kimwipe. Immediately after amputation, PT fragments were transferred into octanol (8-OH) solution (Sigma Aldrich, RM00050 (St. Louis, MO, USA)), and kept in sterile, deep-dish petri dishes (Fisherbrand, 100 mm × 20 mm). Octanol solution was prepared by slowly pipetting 8 µL 8-OH into 500 mL Poland Springs water, resulting in a final concentration of 123 µM, and allowing the solution to vortex for >30 min. Fragments were then left to regenerate in 8-OH for three days, with daily refreshment of the drug. After three days, fragments were transferred into fresh water until regeneration was complete enough to score head morphology (~10 days).

### 4.2. Morphometrics

Worms used for morphometric analysis were imaged using a Nikon SMZ1500 microscope (Melville, NY, USA) with a Retiga 2000R camera (Surrey, BC, Canada) and Q-Capture imaging software (Surrey, BC, Canada). Care was taken to image worms when their heads were most relaxed, in order to capture morphology as accurately as possible (all worms were imaged prior to fixation, which can distort the heads’ external edges). Landmark data were then recorded using ImageJ (Bethesda, MD, USA) [172]. Landmarks for morphometric analysis were chosen based on biological relevance, and reproducibility across worms with varying head morphologies. Landmarks are: (1) the anterior tip of the head; (2) and (3) along the lateral edges at a distance of 1/6th of the length of the worm from the anterior tip of the worm; (4) and (5) the plane of the eyes; (6) and (7) the widest point of the head (often the auricles, if present); (8) and (9) halfway between the eyes and the anterior tip of the head; and (10) and (11) the thinnest point of the head posterior to landmarks 6 and 7. *n* = 10 WT (wild-type) *G. dorotocephala*, *n* = 9 WT *D. japonica*, *n* = 6 WT *P. felina*, *n* = 8 WT *S. mediterranea*, *n* = 7 pseudo *G. dorotocephala*, *n* = 13 pseudo *D. japonica*, *n* = 5 pseudo *P. felina*, and *n* = 6 pseudo *S. mediterranea* flatworms were measured. For measurements of brain in immunohistochemistry, the posterior edge of the brain was consistently defined as the place where nervous tissue enlarged relative to the ventral nerve cord. MorphoJ software (Manchester, UK) [173] was used for Canonical Variate Analysis, in order to quantify and graphically represent changes in head morphology. MorphoJ software was also used to calculate Procrustes distances, and perform statistical analyses.

### 4.3. Visualization of Relative Membrane Potentials with DiBAC

DiBAC4(3) (*bis*-[1,3-dibarbituric acid]-trimethine oxanol) (Invitrogen, Carlsbad, CA, USA) was used as previously [79,83]. A stock solution (1.9 mM) was diluted 1:1000 (0.19 uM) in Poland Springs water, and worms were soaked in the DiBAC solution for >30 min before imaging. Worms were then immobilized in 2% low-meting point agarose, using custom-fabricated Planarian Immobilization Chips as in [174]. Images of the ventral side of immobilized planaria were captured with the Nikon AZ100 Stereomicroscope, Melville, NY, USA, using epifluorescence optics, and NIS-Elements imaging software (Melville, NY, USA). Data were neither altered nor removed.

### 4.4. Immunohistochemistry

Whole worm immunohistochemistry was done as in [175]. Primary antibodies were 3C11 anti-synapsin raised against mouse (used at 1:50 dilution), obtained from the Developmental Studies Hybridoma Bank, created by the NICHD of the NIH and maintained at the University of Iowa, Department of Biology, and α-phosphorylated histone H3 (H3P) 1:250 (Upstate). Secondary antibody was goat anti-mouse Alexa488 (Sigma, St. Louis, MO, USA), used at 1:400 dilution (for anti-synapsin), and an HRP-conjugated anti-rabbit antibody with TSA-Alexa568 and anti-HRP (Molecular Probes, Eugene, OR, USA) (for anti-H3P).

### 4.5. Voltage Dye Image Analysis

In order to estimate the degree of gap junctional connectivity, we processed voltage dye images to determine the number of isopotential cell fields in MATLAB (MathWorks Inc., Natick, MA, USA). Images were flat-field corrected in Image J (Bethesda, MD, USA). For each image, the area of the flatworm was found by taking the average intensity of all of the pixels in the entire image. The flatworm pixel area was used to calculate five intensity clusters of isopotential for the worm body. Each pixel of the flatworm was categorized into the closest of the five clusters by Euclidean distance. For each image, all of the pixels categorized into one intensity cluster were isolated by thresholding, regions of size less than 15 pixels were removed, and any image holes were filled. The boundaries of the region were then calculated using the Moore-Neighborhood tracing algorithm with a modified Jacob’s stopping criteria (MATLAB function bwboundaries) [176]. The regions from each intensity cluster for each flatworm were summed to compute the total number of regions in an image (Figure 6I).

### 4.6. Modeling

Numerical simulations were carried out with an agent-based model where cells are considered as mass points interacting with each other. Some cells have fixed positions and some cells can move. The forces acting between cells depend on the distance between them. Cell motion is calculated as the motion of their centers according to Newton’s second law. The forces acting on cells include friction/adhesion and pressure/chemotaxis from other cells. Cells can be added, moved, and activated/deactivated by the user to create the initial configuration and during a simulation, so any initial cell configuration can be established. The membrane configuration is fixed during a simulation. Figure S2 contains additional details about the modeling. Numerical modeling was carried out with original C++ code developed for this work. The new software we developed includes a user interface with parameter windows and a computational domain (Figure S3).

## 5. Conclusions

Morphological remodeling poses immense computational challenges for the multicellular organism. Cellular state must be integrated and communicated across time and anatomical distance; parameters of regenerative or developmental programs must be set, and these programs must be implemented to narrow tolerances. While the genome is the source of all structural material in the organism, gap junctions provide unique conduits for using physiological and bioelectrical signals to link cells into information-processing networks, and are therefore ideal complements to genetically-encoded programs. We show that a relatively simple physiological perturbation can derive distinct species-specific morphologies from the same genome, highlighting an important layer of control between the genetics and the anatomy of an organism. Further work is needed to better characterize both the dynamics of these networks and the molecular mechanisms that they inform, as well as develop tools with which to control morphology in experimental contexts and biomedical applications.

## Supplementary Materials

Supplementary materials can be found at: http://www.mdpi.com/1422-0067/16/11/26065/s1.
