# Supplementary material for: Gap Junctional Blockade Stochastically Induces Different Species-Specific Head Anatomies in Genetically Wild-Type Girardia dorotocephala Flatworms
Source: Int J Mol Sci. 2015 Nov 24;16(11):27865–96. doi: 10.3390/ijms161126065 (PMC4661923; doi:10.3390/ijms161126065)
Supplement: Supplementary file 1 [file ijms-16-26065-s001.pdf]

Supplementary Materials: Gap Junctional Blockade Stochastically Induces Different Species-Specific Head Anatomies in Genetically Wild-Type *Girardia dorotocephala* Flatworms

Maya Emmons-Bell, Fallon Durant, Jennifer Hammelman, Nicholas Bessonov, Vitaly Volpert, Junji Morokuma, Kaylinnette Pinet, Dany S. Adams, Alexis Pietak, Daniel Lobo and Michael Levin

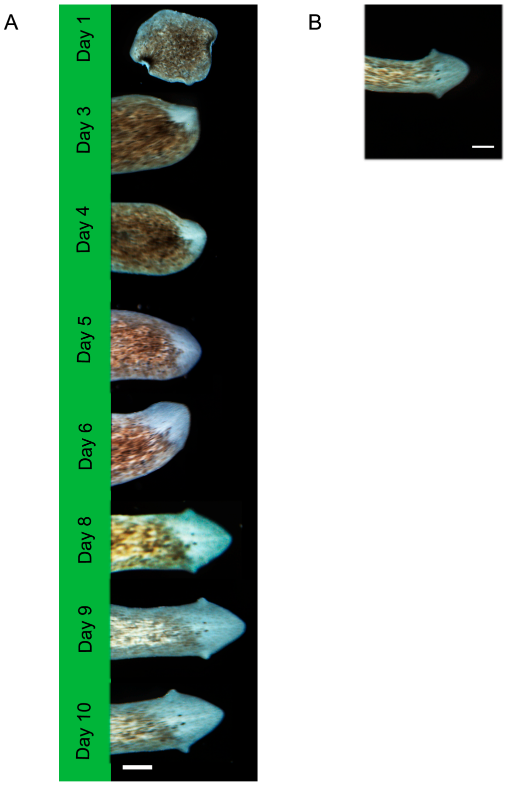

Figure S1. Timeline of regeneration in *G. dorotocephala* flatworms, and 6-OH control. (**A**) Adult morphology is recapitulated by day 10 of regeneration in water (confirmed quantitatively by morphometric analysis in Figure 2. Scale bar 0.5 mm; (**B**) WT GD worms amputated and treated in hexanol for three days, which does not block gap junction channels, and then allowed to regenerate in water for 7 days, show normal regeneration at day 10. Scale bar 0.5 mm.

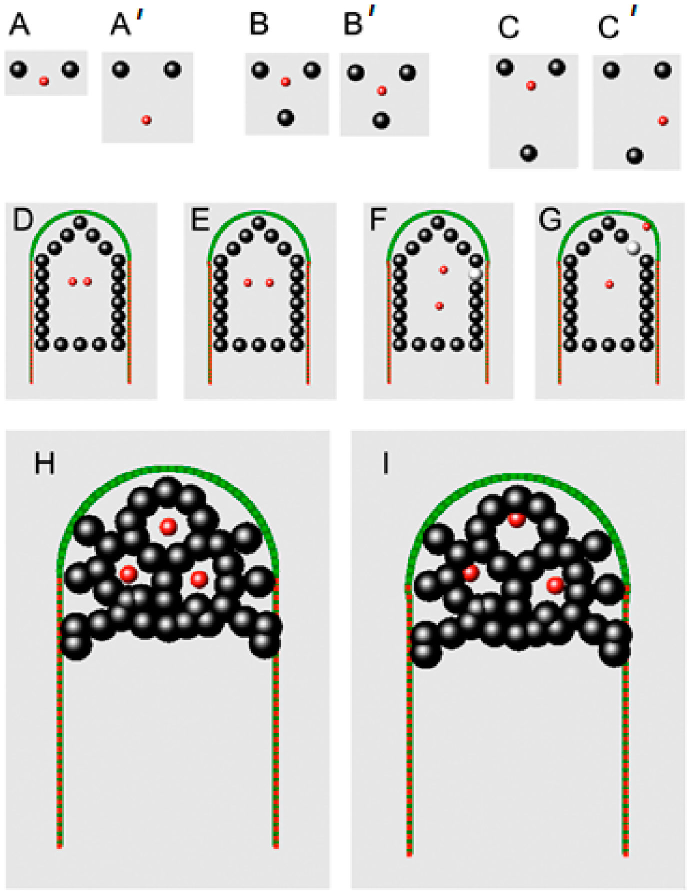

Figure S2. An agent-based model of cell behavior during head patterning. Black cells are fixed and produce substance C. Red cell moves against the gradient of the concentration. In the three examples, there is an initial cell configuration (**A–C**) and a snapshot of cell position some time later (**A′–C′**). In **A**, red cell moves away from the black cells; In **B**, it arrives to its equilibrium position and stays there. In C, the red cell escapes the triangle formed by the black cells and moves away; (**D–G**) Example of cell configuration surrounded by the elastic membrane (see text for details). Cells of type A are shown in black, deactivated cells of type A white, cells of type B are red. The initial cell configuration is shown in panel **H**, while their equilibrium position is shown in panel **I**.

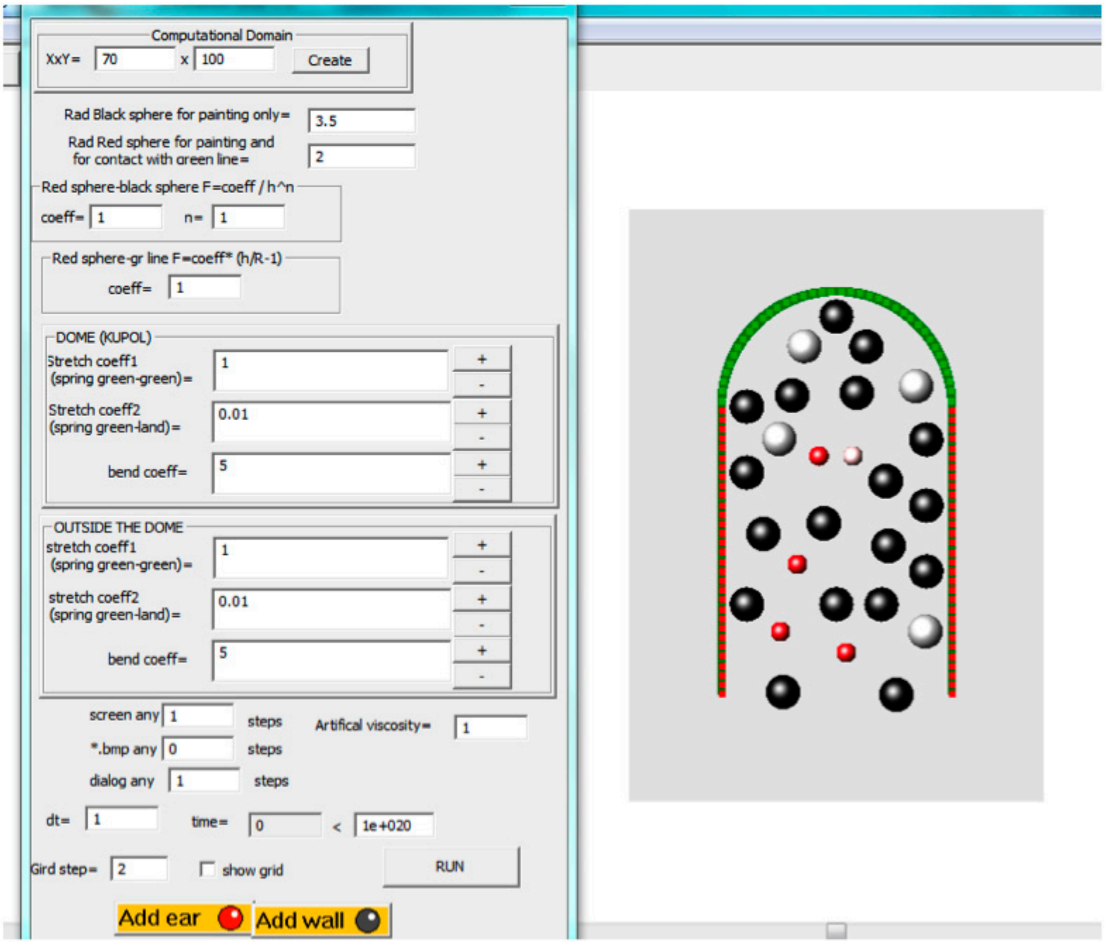

Figure S3. User interface of the software implementing a quantitative model of morphogenesis. The software implements an agent-based model of cell movement and the resulting morphogenesis of a planarian head. This screenshot shows the user interface with a parameter window (**left**) and the resulting computational domain (**right**).
